# Nanodosimetry-Based Plan Optimization for Particle Therapy

**DOI:** 10.1155/2015/908971

**Published:** 2015-06-08

**Authors:** Margherita Casiraghi, Reinhard W. Schulte

**Affiliations:** ^1^Center for Proton Therapy, Paul Scherrer Institut (PSI), 5232 Villigen, Switzerland; ^2^Division of Radiation Research, Loma Linda University, Loma Linda, CA 92350, USA

## Abstract

Treatment planning for particle therapy is currently an active field of research due uncertainty in how to modify physical dose in order to create a uniform biological dose response in the target. A novel treatment plan optimization strategy based on measurable nanodosimetric quantities rather than biophysical models is proposed in this work. Simplified proton and carbon treatment plans were simulated in a water phantom to investigate the optimization feasibility. Track structures of the mixed radiation field produced at different depths in the target volume were simulated with Geant4-DNA and nanodosimetric descriptors were calculated. The fluences of the treatment field pencil beams were optimized in order to create a mixed field with equal nanodosimetric descriptors at each of the multiple positions in spread-out particle Bragg peaks. For both proton and carbon ion plans, a uniform spatial distribution of nanodosimetric descriptors could be obtained by optimizing opposing-field but not single-field plans. The results obtained indicate that uniform nanodosimetrically weighted plans, which may also be radiobiologically uniform, can be obtained with this approach. Future investigations need to demonstrate that this approach is also feasible for more complicated beam arrangements and that it leads to biologically uniform response in tumor cells and tissues.

## 1. Introduction

Particle therapy is becoming increasingly more common for the treatment of cancer. Charged particles have advantages compared to photon therapy due the favorable depth-dose distribution (Bragg peak). Protons, to some degree, and heavy ions, in particular, are characterized by enhanced biological effectiveness in the Bragg peak. While this feature may be useful for the treatment of radioresistant and hypoxic tumors, it also poses a challenge: treatment planning for ion beams is based on the absorbed dose multiplied by the relative biological effectiveness (RBE). This quantity depends on many physical and biological factors, for example, particle type, linear energy transfer (LET), cell type, and biological endpoint and it is therefore difficult to determine and subject to uncertainties.

In proton therapy, the current clinical practice is to apply a constant generic RBE value of 1.1, neglecting the tendency of larger RBE in the distal part of the spread-out Bragg peak (SOBP). Although there are no firm clinical data indicating that this practice should be changed, one can expect based on clinical evidence reviewed in [1] that the RBE of clinical proton beams is depth dependent. On average, there will be an increase in RBE of ≈5% at 4 mm and ≈10% at 2 mm proximal to the distal edge of the SOBP, relative to the RBE at the midpoint of the SOBP: but the number of animals required to estimate this variation to within a few percent* in vivo* would be too large to be practical. A higher RBE can be expected for the stopping low energy protons on the distal fall-off of the SOBP, where the dose decreases rapidly, effectively extending the clinically significant dose range by 1-2 mm. Nevertheless, this uncertainty of RBE can have clinical consequences due to the hesitance of the clinician to aim the beam at organs at risk, or, if he or she does due to unexpected side effects. RBE variations are substantially larger in heavy ion therapy and can not be neglected. In this case, the RBE dependence on dose, type of tissue, and LET are included in the treatment planning process. The current practice is to calculate a depth-dependent RBE value using a biophysical model and adjust the absorbed dose in order to ensure uniform biological effectiveness at each position of the SOBP. Different particle treatment centers use either one of the three biophysical models available to derive biological weighting factors, that is, the local effect model (LEM), the microdosimetric kinetic model (MKM), or the semiempirical passive scattering model [2]. Each model introduces a set of different parameters, which are extracted form experimental data. The use of different models and different delivery modalities produces differences in the estimated biological weighted dose up to 20%, making it difficult to compare clinical results from different centers [3]. Since the present approaches are obviously insufficient for providing a satisfactory method to equalize biological effectiveness at each position of the SOBP, a new system of measurable radiation quality descriptors is needed [4, 5].

It is generally accepted that radiation track structure, that is, the geometric distribution of energy transfer points, is an important factor in the formation of initial DNA damage and its repairability. The local clustering of individual transfer points, in particular ionizations, appears to be important for the production of double strand breaks (DSBs) of different degrees of complexity [6, 7]. Large ionization clusters occurring in a DNA molecule and surrounding water are believed to be a major source of unrepaired DSBs, which will determine the ultimate fate of the cells and tissues harboring them. Regional clustering of DSBs within chromosomal domains may, in addition, decrease their repairability [8]. Monte Carlo (MC) track structure codes for detailed studies of radiation interaction with DNA have been developed and are reviewed in [9]. More recently, Geant4-DNA, an extension of the Geant4 MC toolkit has been released [10] for calculation of track structure at the nanometric level.

In addition to theoretical track structure and DNA damage studies, experimental methods have been developed to measure the distribution of ionization clusters in DNA-like cylindrical sensitive volumes (SVs) using nanodosimetery [11]. One vision for the future is that nanodosimetric ionization cluster size distributions (ICSDs) and related statistical parameters may serve as descriptors of radiation quality with biological relevance. These quantities may be simulated with dedicated MC track structure codes which will be benchmarked (for the gas phase) with nanodosimetric measurements. This opens the possibility to use nanodosimetric descriptors as the basis for the treatment plan optimization in particle therapy or quality factors for mixed radiation fields in radiation protection applications.

The goal of this work was to perform a preliminary study to test the feasibility of a novel approach to optimize biologically-weighted intensity modulated particle treatment planning using nanodosimetric quantities. The basic idea behind this approach is optimize fluences of individual pencil beams (PB) in order to create a mixed radiation field with equal nanodosimetric descriptors at each position of the SOBP. Simple proton and carbon treatment plans with single- or two-opposing field were simulated in a water phantom with Geant4 and nanodosimetric parameters at many positions throughout the target volume were calculated. Under the reasonable assumption that nanodosimetric descriptors of radiation quality are related to the initial DNA damage, this approach may produce optimized biologically-weighted treatments for intensity modulated charged particle therapy.

## 2. Materials and Methods

### 2.1. Monte Carlo Simulations of Treatment Plans

The simulations presented in this work were performed with the Geant4 Monte Carlo toolkit version 10.00 [12], including the Geant4-DNA extension [10].

**Figure 1:**
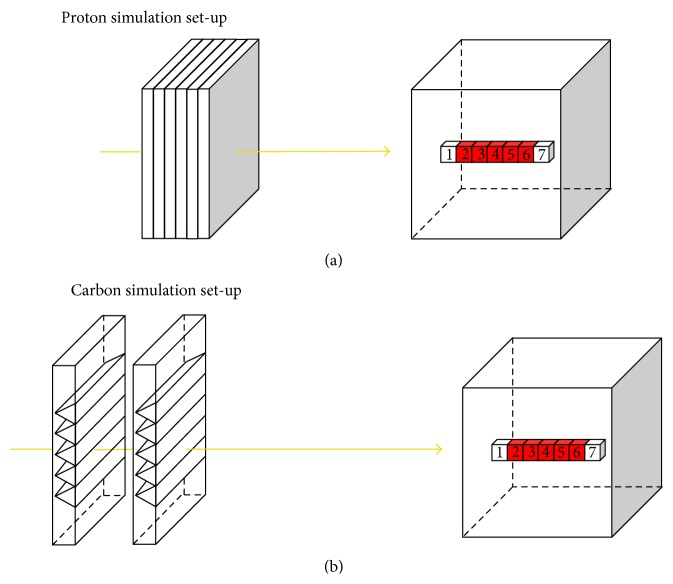
Sketch of the simulation set-up. Top: proton simulations with range shifters and water phantom shown. Bottom: carbon simulations with two ripple filters and water phantom shown. Red-colored voxels represent the target region.

In order to prove the principle of the proposed optimization method, simple linear target geometry and beam arrangement were simulated for the proton and the carbon ion plans (Figure 1). A target volume, comprising a row of five cubic voxels of 5 mm side length, was created inside a cubic water phantom of 20 cm side length. To introduce a nonsymmetric target position, the target center was shifted in beam direction by 3.75 cm from the phantom center. As a consequence, the target was at 5 cm and 9.5 cm from the left and right sides of the phantom, respectively. Two additional voxels, were added at the right and left edges of the target to get dosimetry information outside the target volume, that is, in normal tissue. Beam arrangements with either a single-field or two-opposing fields impinging on the lateral aspect of the phantom were simulated. Each field consisted of ten monoenergetic Gaussian pencil beams (PB) with a size (sigma) of 3 mm. The energies of the PBs where chosen from a calibration curve of Bragg peak depth in water versus PB energy in order to create a SOBP with Bragg peak spacing of 2.5 mm in depth, that is, two PBs aimed at each target voxel for single-field plans. In order to achieve an acceptable homogeneity of the SOBP, range shifters and ripple filters were included in the simulations. In the proton plan simulations, twenty-four polyethylene range shifters of 0.45 cm thickness each were placed along the beam, at 10 cm from the phantom surface (Figure 1(a)). The PB energy selected in order to cover the target ranged from 151 MeV to 186 MeV. In the carbon ion plan simulations, two ripple filters designed as in [13] were placed along the beam path, at 35 cm from the phantom surface (Figure 1(b)). PB energies ranged from 154 MeV/u to 246 MeV/u.

**Figure 2:**
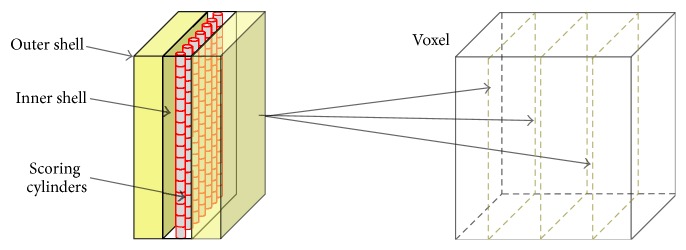
Sketch, not in scale, of the microscopic simulation geometry. Slabs comprising an outer shell, an inner shell, and a region with scoring cylinders were placed at three positions in each voxel.

The Geant4-DNA extension was used in the simulation of radiation track structure. Using this extension, protons, carbon ions, and their secondaries were transported step-by-step in water down to very low energies. A cut-off of 11 eV was set for electrons. The spatial distribution of the ionization events was obtained from the simulations. The step-by-step tracking is expensive in computation time. Therefore, the target was divided into a series of subvolumes, defined as regions (Figure 2). The step-by-step transportation was activated in the scoring volume plus a surrounding shell of micrometric thickness, while the standard Geant4 condensed history transportation with region specific production cuts was used for the rest of the volumes. In the world and water phantom regions, the Livermore electromagnetic (EM) physics models were activated and a production cut of 100 *μ*m was set for secondaries. As shown in Figure 2, three slabs of 5 × 5 mm^2^ area and 20 *μ*m thickness (outer shell) were placed in each of the five target voxels and in the two normal tissue voxels at three different locations (proximal, center, and distal). The Livermore EM models with a production cut of 1 *μ*m were used in the outer shell regions. The Livermore EM models with a production cut of 10 *μ*m were used in the rest of the voxel volume. An additional region (inner shell) comprising a volume of 5 × 5 mm^2^ area and 2 *μ*m thickness was embedded at the center of each outer shell. In these volumes, the step-by-step transportation using the DNA EM models was activated. Finally, a two-dimensional array of 10^4^ × 10^4^ cylindrical volumes of 500 nm diameter and 500 nm height was created at the center of each inner shell region and set as scoring volume (scoring cylinders). The *x*, *y*, and *z* coordinates of the ionization events produced by the primary particles and their secondaries were collected in the scoring cylinders. The hadron physics models and radioactive decay physics were activated in addition to the EM physics models in order to take into account nuclear interactions.

For the proton plans, 10^5^ histories per PB were simulated, while for the carbon ion plans 5 × 10^4^ histories were simulated due to the larger computation time necessary for carbon tracking. The calculation time for the entire plan was of the order of days for protons of weeks for carbon ions.

### 2.2. Calculation of Nanodosimetric Quantities

The quantity measured in nanodosimetry is the ionization cluster size (ICS), defined as the number of ionizations produced in a given SV per primary particle. This quantity is stochastic and is characterized by a distribution. The ICSD is defined as the probability distribution *P*(*ν*∣*Q*) of the number of ionizations *ν* generated in a SV per primary particle of radiation quality *Q*. The ICSD is characteristic of the unique track structure of a specific radiation quality for a given SV. This distribution includes the contribution from the secondary particles and depends on the geometric characteristics of the SV. In MC simulations of ICSD, the SV is usually chosen to be a water cylinder of nanometric dimensions assumed to represent a DNA short segment [14].

**Figure 3:**
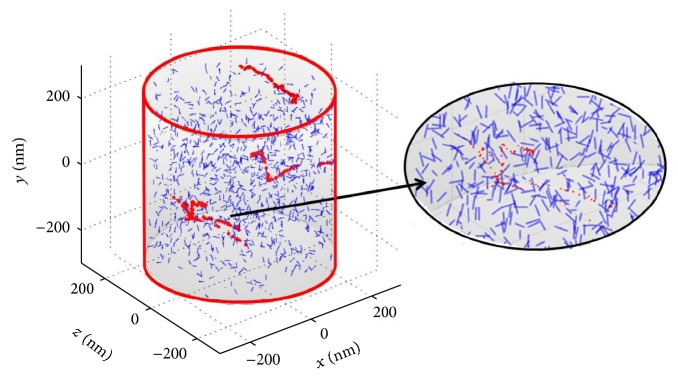
Representation of the track sampling procedure. The big cylinder represents one of the scoring cylinders. Small cylinders with random position and orientation were the SVs used for track sampling.

The radiation tracks obtained in the simulations of this work were analyzed with cylindrical SV of 2 nm diameter and 16 nm length (approximately 50 base pairs) placed at random position and orientation within the scoring volumes (Figure 3). Each scoring cylinder was sampled with 10^4^ random cylinders, and the number of ionizations collected in each SV was stored for the ICSD computation.

For each pencil beam *i* and voxel *j*, the following ICSDs were computed.

- *Absolute ICSD*, *P*_*i*,*j*_(*ν*∣*Q*), represents the probability of generating a cluster of *ν* ionizations normalized to the total number of initial primary particles. That is, events producing zero ionizations in the SV are included in the computation. *Q* represents the radiation quality of the radiation field produced by the PB *i* in the voxel *j*.
- *Conditional ICSD*, *P*_*i*,*j*_^*∗*^(*ν*∣*Q*), represents the probability of generating a cluster of *ν* ionizations normalized to the number of primary particles generating at least one ionization in the SV.

ICSDs were computed at three depths (proximal, central, and distal) in each voxel and the results were averaged in order to obtain a representative ICSD for PB *i* and voxel *j*.

From the absolute distributions, the following nanodosimetric descriptors were derived.

(i) * Mean absolute ion cluster size (ICS)*:
  (1) $$\begin{array}{c} {\left(\;M_{1}\;\right)}_{i,j}=\overset{\infty }{\underset{\nu =0}{\sum }}\nu P_{i,j}\left(\;\nu \;|\;Q\;\right). \end{array}$$
 This quantity is the first moment of the absolute ICSD and represents the mean number of ionizations produced in the SV by the PB *i* in voxel *j*.
(ii) * Yield of small clusters*:
  (2) $$\begin{array}{c} {\left(\;Y_{SC}\;\right)}_{i,j}=\overset{3}{\underset{\nu =2}{\sum }}P_{i,j}\left(\;\nu \;|\;Q\;\right). \end{array}$$
 Small clusters were defined as those clusters comprising two or three ionizations. Such clusters are generally assumed to be responsible for isolated or simple (no additional damage) DSBs.
(iii) * Yield of large clusters*:
  (3) $$\begin{array}{c} {\left(\;Y_{LC}\;\right)}_{i,j}=\overset{10}{\underset{\nu =4}{\sum }}P_{i,j}\left(\;\nu \;|\;Q\;\right). \end{array}$$
 Large clusters were defined as those clusters comprising four to ten ionizations. Such clusters are generally assumed to be responsible for complex DSBs or other complex damages. Very large clusters with more than ten ionizations were not included in the optimization.

The listed nanodosimetric quantities were calculated for unit PB fluence, that is, one primary particle per PB, and were used as starting conditions for the optimization of the treatment plans.

Furthermore, the following quantities were calculated for voxel *j* in order to evaluate the radiation quality of the composite radiation fields contributed by all PBs.

(i) * Mean conditional ICSD*:
  (4) $$\begin{array}{c} {\left(\;M_{1}^{*}\;\right)}_{j}=\frac{\sum_{\nu =1}^{\infty }\nu P_{j}^{*}\left(\nu \;|\;Q\right)}{\sum_{\nu =1}^{\infty }P_{j}^{*}\left(\nu \;|\;Q\right)}, \end{array}$$
 where *P*_*j*_^*∗*^(*ν*∣*Q*) = ∑_*i*=1_^#PBs^ *P*_*i*,*j*_^*∗*^(*ν*∣*Q*).
 This quantity is the fist moment of the conditional ICSD *P*_*j*_^*∗*^(*ν*∣*Q*) produced by the mixed field generated by the contribution of all PBs in the voxel *j*.
(ii) *Biologically effective mean ICS:*
  (5) $$\begin{array}{c} {\left(\;M_{1}^{bio}\;\right)}_{j}\left(\;Q\;\right)=\frac{\sum_{\nu =2}^{10}\nu P_{j}^{*}\left(\nu \;|\;Q\right)}{\sum_{\nu =2}^{10}P_{j}^{*}\left(\nu \;|\;Q\right)}. \end{array}$$
 This quantity was defined for evaluating the radiation quality of the mixed radiation field in terms of clusters with 2–10 ionizations.

The maximum statistical error was less than 1% for all nanodosimetric quantities.

### 2.3. Plan Optimization

The* MATLAB* function* lsqlin* was used to solve the constrained linear least-squares problem associated with the PB fluence optimization. Two different optimization strategies with different goals were used.

- *Uniform M*_1_: the beam PB fluences *w*_*i*_ were optimized with the goal to obtain a uniform mean absolute ionization yield in the target.
- *Uniform cluster yields*: the beam PB fluences *w*_*i*_ were optimized with the goal to obtain uniform yields of small and large clusters in the target.

The first optimization strategy is equivalent to achieving a uniform absorbed dose plan. The mean ionization yield *M*_1_ is related to absorbed macroscopic dose according to (6), where *W* is the mean energy required to form an ion pair upon the complete the slow down of ionizing particles and *m* is the SV mass

(6) $$\begin{array}{c} D=\frac{M_{1}W}{m}\left[\;Gy\;\right]. \end{array}$$

The optimization of single- and two-field proton and carbon ion plans were analyzed in two steps. First, conditional and absolute ICSDs produced in the irradiated volume were calculated in each voxel of interest for unit fluence. Secondly, PB fluences were optimized to obtain either uniform *M*_1_ or uniform cluster yields, and the optimized plans were evaluated in terms of nanodosimetric quantities.

## 3. Results

### 3.1. Proton Plans

#### 3.1.1. Single-Field Optimization

**Figure 4:**
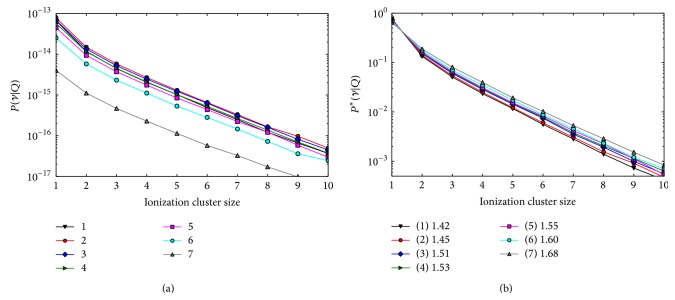
Composite ICSDs obtained in the five target voxels (2–6) and in the two normal tissue voxels (1 and 7) for the single-field proton plan with unit PB fluence. Left panel: absolute distributions. Due to the the large frequency of zero clusters, the zero bin is not shown for a better visualization of the plot. Right panel: conditional distributions. In the legend box, the left panel shows the coding of the corresponding voxels (refer to Figure 1 for the voxel numbering scheme). The legend box in the right panel also shows the *M*_1_^*∗*^ for each voxel.

In Figure 4, the composite ICSDs obtained for the single-field proton plan with PB unit fluences are shown. The plots in the left panel represent the composite absolute ICSDs produced by all PBs in the five target voxels (2–6) and in the two normal tissue voxels (1 and 7). Due to the single-field arrangement, the fluence decreases with depth, which leads to lower absolute frequencies as seen in the plot. The probability to obtain one or more ionization in the SV is larger for the superficial voxels of the target and is lower in the most distal voxel, where only the tail of the most penetrating Bragg peak is present. In the right panel, the composite conditional ICSDs are shown. The conditional ICSDs and their first moments *M*_1_^*∗*^ are representative of the radiation quality present in each voxel. The larger clusters occur with larger frequencies in the distal voxels (6 and 7) where the dose is delivered exclusively by stopping protons. *M*_1_^*∗*^ increases with the increasing depth; for example, *M*_1_^*∗*^ is 18% larger in the distal voxel than in the entrance voxel. This behavior is due to the increasing contribution of stopping protons towards the distal end of the field.

**Figure 5:**
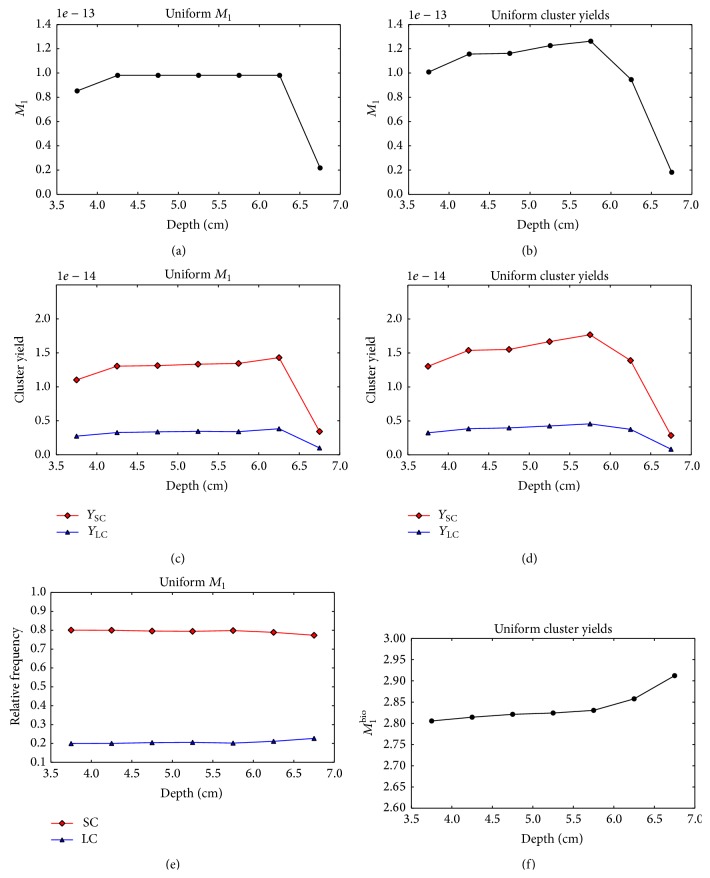
Nanodsimetric quantities calculated for the optimized single-field proton plans as a function of the voxel depth. Results of the* uniform M*_1_ optimization and* uniform cluster yield* optimization are shown in the left and right columns, respectively. (a), (b) Composite *M*_1_. (c), (d) Composite *Y*_SC_ (squares) and *Y*_LC_ (triangles). (e) Frequency of small clusters *Y*_SC_ (squares) and large clusters *Y*_LC_ (triangles) relative to the total yield *Y*_SC_ + *Y*_LC_. (f) Composite *M*_1_^bio^.

Figure 5 shows the composite *M*_1_, *Y*_SC_, and *Y*_SC_ values for the two PB fluence optimization strategies as a function of voxel depth. As expected, the* uniform M*_1_ optimization strategy produces a uniform *M*_1_ distribution in the target (Figure 5(a)). However, the yields of both small and large clusters increase with depth (Figure 5(c)). In Figure 5(e), the frequencies of small and large clusters relative to the total yield of clusters produced in the voxels are shown. The relative frequency of large clusters slightly increases with depth and a steeper increase is observed in the last two voxels, where the contribution of low energy proton increases. For the* uniform cluster yield* optimization strategy, the optimization algorithm decreases the small and large cluster yields in the distal voxels assigning lower weights to the most penetrating PBs (Figure 5(d)). However, an optimal solution providing a uniform yield of both large and small clusters could not be found due to the insufficient number of degrees of freedom for this optimization problem.  Figure 5(f) shows that *M*_1_^bio^ increases with the increasing depth despite the PB fluence optimization.

#### 3.1.2. Two-Opposing Field Optimization

**Figure 6:**
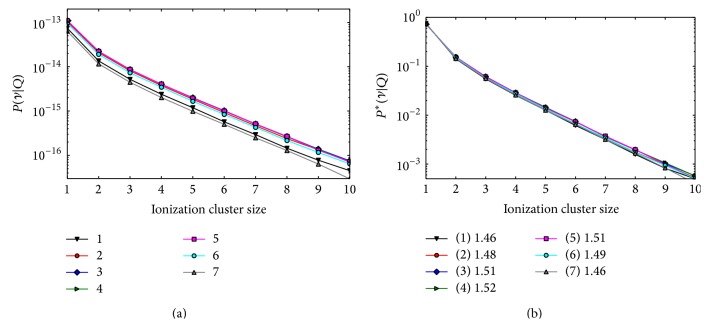
Composite ICSDs obtained in the five target voxels (2–6) and in the two normal tissue voxels (1 and 7) for the two-field proton plan with unit PB fluence. Left panel: absolute distributions (frequencies of zero clusters not shown). Right panel: conditional distributions. The voxel numbering scheme is the same as Figure 4. The legend box, in the right panel shows the *M*_1_^*∗*^ values for each voxel.

In Figure 6, the composite ICDSs obtained for a proton plan with two-opposing fields using unit PB fluences are shown. The absolute ICSDs (left panel) have similar frequencies for all the target voxels and the ionization frequency is smaller in normal tissue voxels where a lower particle fluence is present and the radiation field is mainly comprised of fast protons. The conditional ICSDs (right panel) are almost overlapping, indicating that the mixed field radiation quality is similar at all the target depths. *M*_1_^*∗*^ in the normal tissue voxels at the target edge has the same values as in the target due to the distal Bragg peak penumbra of the most penetrating PBs.

**Figure 7:**
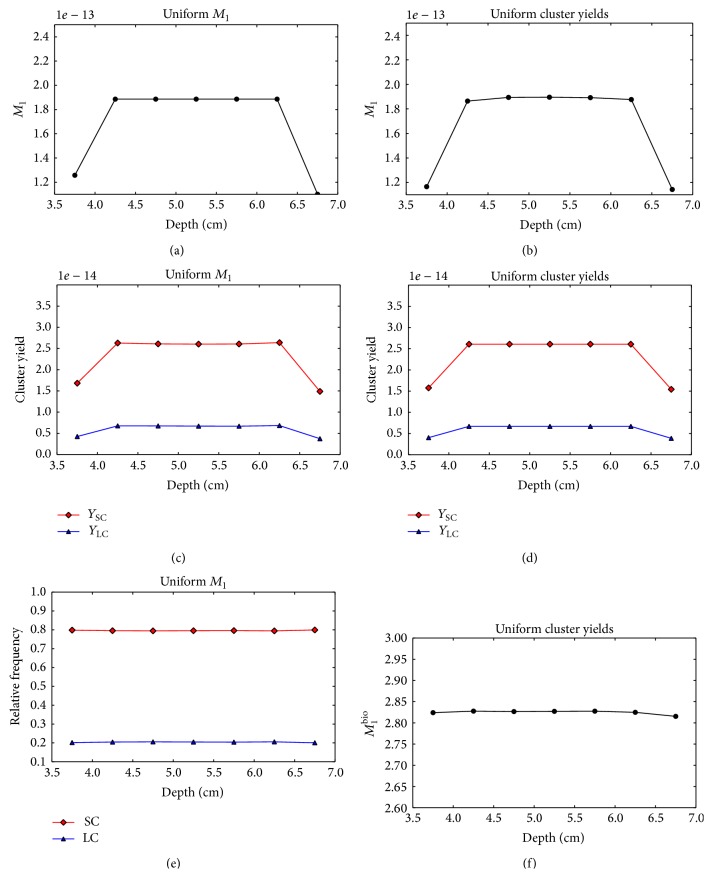
Nanodsimetric quantities calculated for the optimized two-field proton plans as a function of the voxel depth. Results of the* uniform M*_1_ optimization and* uniform cluster yield* optimization are shown in the left and right columns, respectively. (a), (b) Composite *M*_1_. (c), (d) Composite *Y*_SC_ (squares) and *Y*_LC_ (triangles). (e) Frequency of small clusters *Y*_SC_ (squares) and large clusters, *Y*_LC_ (triangles) relative to the total yield *Y*_SC_ + *Y*_LC_. (f) composite *M*_1_^bio^.

Both the* uniform M*_1_ and* uniform cluster yield* optimization were successful in this case. Figures 7(c) and 7(d) show that uniform distributions of both *Y*_SC_ and *Y*_LC_ are obtained in the optimized plans. This result is due to the opposing beam configuration. The dose is delivered by the the same number of Bragg peaks and plateaus in each target voxel. Therefore, radiation fields of similar quality are present at all depths. The relative frequency of small and large clusters displayed in Figure 7(e) confirms that all the voxels are irradiated with the the same share of densely and sparsely ionizing radiation. The distribution of *M*_1_^bio^ is also uniform in the target and it slightly decreases in the normal tissue voxels (Figure 7(f)).

### 3.2. Carbon Ion Plans

#### 3.2.1. Single-Field Optimization

**Figure 8:**
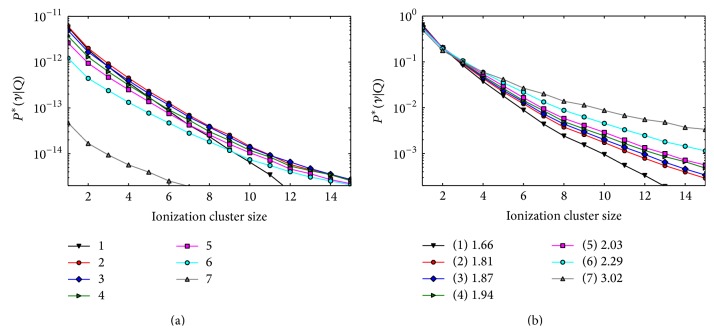
Composite ICSDs obtained in the five target voxels (2–6) and in the two normal tissue voxels (1 and 7) for the single-field carbon plan with unit PB fluence. Left panel: absolute distributions (frequencies of zero clusters not shown). Right panel: conditional distributions. The voxel numbering scheme is the same as Figure 4. The legend box, in the right panel shows the *M*_1_^*∗*^ values for each voxel.

Figure 8 shows the composite ICSDs obtained for the single-field carbon ion treatment plan. The absolute ICSDs (left panel) show a larger frequency of all clusters per unit particle fluence compared to protons. The densely ionizing effect of carbon ions is apparent in the conditional ICSDs plots (right panel) demonstrating much larger relative frequencies of larger ionization clusters and *M*_1_^*∗*^ values compared to protons, in particular in the most distal voxels. A percentage difference of 82% for *M*_1_^*∗*^ is found between the proximal and distal voxel. This is explained by the rapid increase of LET with penetration depth for carbon ions. Carbon ion fragments will also contribute to the increase of *M*_1_^*∗*^ in the distal voxels.

**Figure 9:**
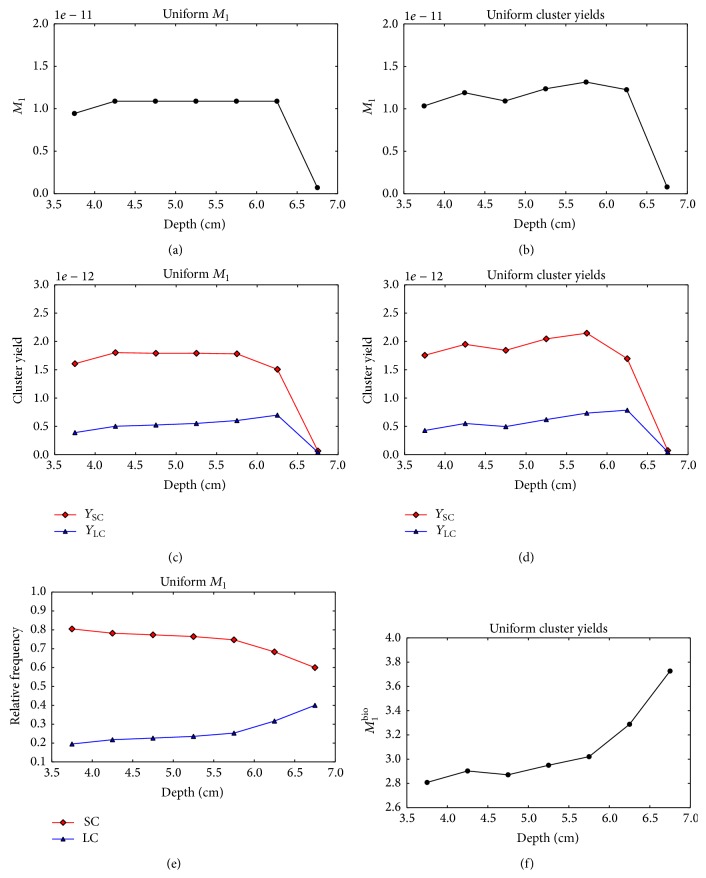
Nanodsimetric quantities calculated for the optimized single-field carbon plans as a function of the voxel depth. Results of the* uniform M*_1_ optimization and* uniform cluster yield* optimization are shown in the left and right columns, respectively. (a), (b) Composite *M*_1_. (c), (d) Composite *Y*_SC_ (squares) and *Y*_LC_ (triangles). (e) Frequency of small clusters, *Y*_SC_ (squares) and large clusters *Y*_LC_ (triangles) relative to the total yield *Y*_SC_ + *Y*_LC_. (f) Composite *M*_1_^bio^.

Figures 9(a) and 9(c) demonstrate that the* uniform M*_1_ optimization of the single-field plan leads to a nonuniform distribution of small and large cluster yields in the target. The small cluster yields decrease with the increasing voxel depth, while the yield of large clusters rapidly increases. The plots in Figure 9(e) show that the relative contribution of small and large clusters varies with depth. As in the case of protons, the optimization of uniform cluster yields does not converge to an acceptable solution (Figures 9(b), 9(d), and 9(f)).

#### 3.2.2. Two-Opposing Field Optimization

**Figure 10:**
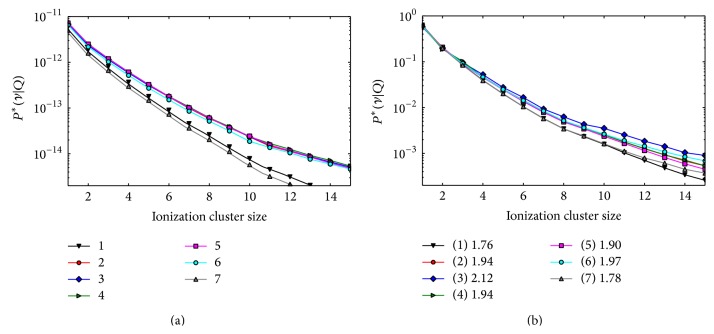
Composite ICSD obtained in the five target voxels (2–6) and in the two normal tissue voxels (1 and 7) for the two-field carbon plan with unit PB fluence. Left panel: absolute distributions (frequencies of zero clusters not shown). Right panel: conditional distributions. The voxel numbering scheme is the same as Figure 4. The legend box in the right panel shows the *M*_1_^*∗*^ values for each voxel.

As in the case of the proton plan, the two-opposing field beam arrangement produced a more uniform composition of radiation field qualities in all voxels. The ICSDs distributions were similar in all the voxels, although the difference among the curves was larger than that for the two-field proton plan (Figure 10), indicating a larger variation of the radiation quality with depth. The difference between the minimum and maximum *M*_1_^*∗*^ in the target was 11%.

**Figure 11:**
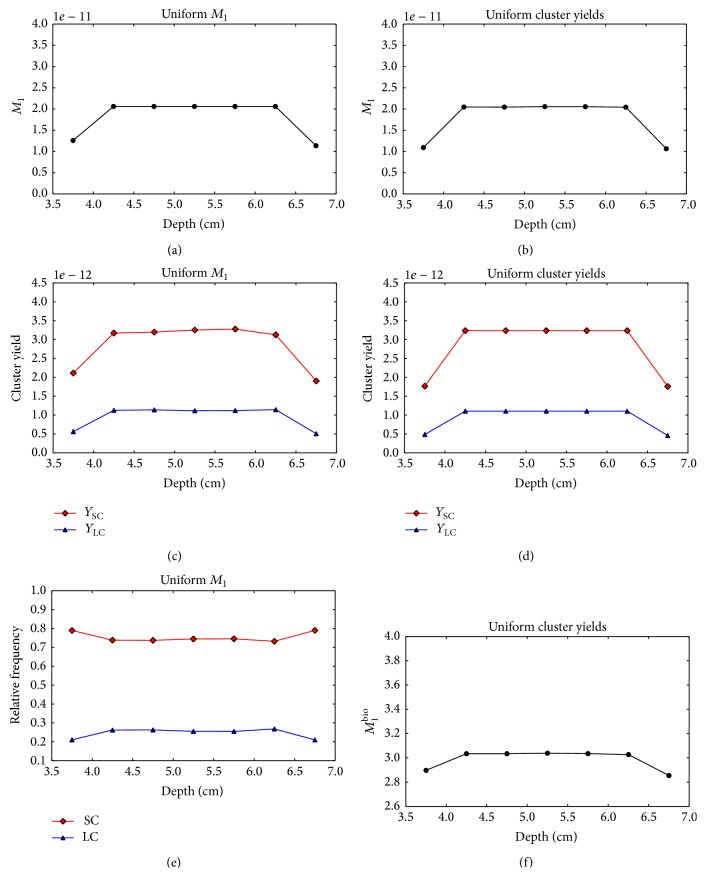
Nanodsimetric quantities calculated for optimized the two-field carbon plans as a function of the voxel depth. Results of the* uniform M*_1_ optimization and* uniform cluster yield* optimization are shown in the left and right columns, respectively. (a), (b) Composite *M*_1_. (c), (d) Composite *Y*_SC_ (squares) and *Y*_LC_ (triangles). (e) Frequency of small clusters *Y*_SC_ (squares) and large clusters *Y*_LC_ (triangles) relative to the total yield *Y*_SC_ + *Y*_LC_. (f) Composite *M*_1_^bio^.

In this case, the* uniform M*_1_^*∗*^ optimization leads to a slightly nonuniform distribution of *Y*_SC_ and *Y*_LC_ in the target as shown in Figures 11(c) and 11(e). On the other hand, the* uniform cluster yield* optimization produces a plan with a flat distribution of both large and small clusters (Figure 11(d)). Although it was not included in the optimization objective, a uniform *M*_1_ distribution was also obtained in this case (Figure 11(b)). The *M*_1_^bio^ calculated for the optimized plan was constant in the target and lower in the normal tissue (Figure 11(f)).

## 4. Discussion

A common approach to take into account the changing biological effectiveness of therapeutic ion beams has been to modify the physical dose according to the RBE concept. This requires knowledge of RBE for relevant target cells and for the dose delivered. Moreover, different biophysical models for calculating RBE such as the LEM or the MKM can lead to differences in the prescribed dose by more than 10%, which is unacceptable. In this work, a novel optimization strategy for particle therapy treatment planning has been proposed. The approach is based on the optimization of nanodosimetric quantities assumed to be related to the radiobiological effect. The nanodosimetry-based optimization is independent of RBE and does not require a specific biophysical model. The approach rather depends on the physical quantities that can be simulated with MC track structure codes and benchmarked with experimental measurements. The feasibility of this planning strategy was tested for simplified proton and carbon ion plans calculated in a water phantom with Geant4 MC simulations.

In the case of the single-field plans, the* uniform cluster yield* optimization approach did not produce acceptable results. This is expected due to the insufficient degrees of freedom of the optimization problem. Due to the beam configuration, the mixed radiation field in the target varies with depth. Only high LET radiation, producing dense clusters, is present at the distal end while a mixture of high and low LET is found in the other voxels. This makes the simultaneous equalization of *Y*_SC_ and *Y*_LC_ at different target depths impossible. The optimization was successful for the two-opposing beam plan. Uniform distributions of *Y*_SC_ and *Y*_LC_ were obtained in the target for both proton and carbon plans. Although not included in the optimization, a uniform *M*_1_ distribution was also obtained. In this case, the beam configuration is favorable for the equalization of the biologically relevant radiation components, since a balanced mixture of high and low LET radiation is present in all the target voxels. This result increases the confidence of the feasibility of the proposed optimization approach and it points out the importance of the beam configuration in the treatment planning of ion beams. The results are valid for the specific simple geometry of the simulated plans. Further testing is necessary to validate this approach for more realistic scenarios with a three-dimensional target geometry and inclusion of tissue heterogeneity. This would produce more complex mixed radiation fields that may challenge the optimization.

The proposed optimization strategy is based on the assumption that nanodosimetric descriptors are directly related to the biological effect. In this work, *Y*_SC_ and *Y*_LC_ were defined as estimators of radiobiological effect and used in the plan optimization. Single ionizations were neglected in the computation of biological damage, as those are assumed to produce isolated DNA damages that are efficiently repaired by the DNA repair system. On the other hand, clusters with two or more ionizations can produce DSBs which are considered potentially irreparable (or lethal) lesions, with reparability decreasing with the increasing degree of lesion clustering [15]. Following these assumptions, *Y*_SC_ may be related to DSBs that are usually repaired, while *Y*_LC_ may be related to more complex DSBs leading to chromosomal aberrations and cell death. Clusters larger than ten ionizations were not taken into account in the optimization due to the low probability of occurrence and because they are assumed to be less effective in producing complex damage due to the recombination of radiation-induced radicals [16].

The size of the SV used for ionization cluster size sampling is a critical parameter in nanodosimetry as ICSDs strongly depend on SV dimensions. A SV diameter of ≈2 nm corresponding to the DNA double helix width was used in previous nanodosimetry works. In this work, a SV length of 16 nm was chosen. Other authors have used a shorter length of 3.4 nm corresponding to the maximum interaction length of individual breaks forming a DSB [14, 17]. However, we believe that biologically relevant large clusters extending beyond 3.4 nm could be excluded due to the short SV length. This is supported by previous work on calculation of quality factors for different types of particles and energies where a length of 16 nm for the SV was found to lead to more realistic results than a short segment of 3.4 nm [18].

The SVs defined in this work consisted of liquid water as surrogate of DNA components. This choice was due to the fact that Geant4-DNA only includes the physics interaction cross sections for water. Theoretical studies have shown considerable differences between electron and proton energy loss in water and DNA [19, 20]. Experimental cross sections of DNA constituent for electron impact have recently been measured [21]. Future Geant4-DNA releases implementing the recent data will allow the calculation of more accurate results.

The accuracy of the ionization cluster yields calculated in this work relies on the physics of interaction models implemented in Geant4-DNA. At the nanometer scale, step-by-step transportation of electrons down to a theoretical limit of zero eV is important. At these low energies (≪100 eV), the cross sections for liquid water are uncertain. Corrections to the plane wave Born approximation, used to calculate excitation and ionization cross sections, have to be applied. Different correction methods are present in literature and used in different MC track structure codes [22]. Semiempirical corrections as described by Emfietzoglou and Nikjoo [23] are implemented in Geant4-DNA. Alternative models for the calculation of the liquid water dielectric response function based on more recent experimental data are available in the literature [22]. The implementation of these models could improve the accuracy of the ionization and excitation cross sections used for the simulations and as a consequence of the ionization cluster yield calculation. The yields obtained in this work may indeed be overestimated; Vassiliev [24] noticed that Geant4-DNA underestimates the *W*-value of electrons.

As pointed out in [25, 26], the validity of the trajectory approach for transport of low energy electrons may not be compatible with the Heisenberg uncertainty principle. Nevertheless, the approach can be applicable under certain conditions as an approximation of quantum multiple scattering.

An alternative approach for including the radiation quality in proton treatment planning has been recently proposed by Giantsoudi et al. [27]. The authors investigated the feasibility of LET-guided plan optimization. Using a multicriteria optimization module, they were able to select among multiple dose optimized plans those producing a favorable LET distribution, thus improving the RBE-weighed dose. Moreover, they suggested a hybrid optimization, including both dose and LET-based objectives. Also, this approach has the advantage of being based on a physical quantity (LET) which can be predicted with MC simulations. On the other hand, the LET is a nonstochastic parameter describing the energy loss per unit path length rather than the stochastic energy loss in subcellular volumes. Thus, it is only an approximation of the underlying physics and can not be directly related to the radiation tack structure and the yield of biologically effective lesions.

## 5. Conclusion

The feasibility of a novel nanodosimetry-based plan optimization approach was investigated in this work. The proposed approach was successful for plans with simplified target geometry and favorable beam arrangement leading to uniform distributions of biologically relevant nanodosimetric parameters for both proton and carbon plans. The next step necessary before the clinical application of this approach is to perform a series of radiobiological experiments to provide a clear evidence of the relation between nanodosimetric quantities and the biological effectiveness in a number of tumor systems both* in vitro* and* in vivo*.
